# Superior infectivity for mosquito vectors contributes to competitive displacement among strains of dengue virus

**DOI:** 10.1186/1472-6785-8-1

**Published:** 2008-02-13

**Authors:** Kathryn A Hanley, Jacob T Nelson, Erin E Schirtzinger, Stephen S Whitehead, Christopher T Hanson

**Affiliations:** 1 Department of Biology, New Mexico State University, Las Cruces, NM 88003, USA; 2 Laboratory of Infectious Diseases, National Institute of Allergy and Infectious Diseases, National Institutes of Health, Bethesda, MD 20892, USA

## Abstract

**Background:**

Competitive displacement of a weakly virulent pathogen strain by a more virulent strain is one route to disease emergence. However the mechanisms by which pathogens compete for access to hosts are poorly understood. Among vector-borne pathogens, variation in the ability to infect vectors may effect displacement. The current study focused on competitive displacement in dengue virus serotype 3 (DENV3), a mosquito-borne pathogen of humans. In Sri Lanka in the 1980's, a native DENV3 strain associated with relatively mild dengue disease was displaced by an invasive DENV3 strain associated with the most severe disease manifestations, dengue hemorrhagic fever/dengue shock syndrome (DHF/DSS), resulting in an outbreak of DHF/DSS. Here we tested the hypothesis that differences between the invasive and native strain in their infectivity for *Aedes aegypti *mosquitoes, the primary vector of DENV, contributed to the competitive success of the invasive strain

**Results:**

To be transmitted by a mosquito, DENV must infect and replicate in the midgut, disseminate into the hemocoel, infect the salivary glands, and be released into the saliva. The ability of the native and invasive DENV3 strains to complete the first three steps of this process in *Aedes aegypti *mosquitoes was measured *in vivo*. The invasive strain infected a similar proportion of mosquitoes as the native strain but replicated to significantly higher titers in the midgut and disseminated with significantly greater efficiency than the native strain. In contrast, the native and invasive strain showed no significant difference in replication in cultured mosquito, monkey or human cells.

**Conclusion:**

The invasive DENV3 strain infects and disseminates in *Ae. aegypti *more efficiently than the displaced native DENV3 strain, suggesting that the invasive strain is transmitted more efficiently. Replication in cultured cells did not adequately characterize the known phenotypic differences between native and invasive DENV3 strains. Infection dynamics within the vector may have a significant impact on the spread and replacement of dengue virus lineages.

## Background

The mechanisms that drive competitive displacement of one species by another have received considerable attention from ecologists in the context of species invasions by free-living organisms [1-7]. Competitive displacement may play an equally important role in the dynamics of emerging infectious diseases. One of several mechanisms of disease emergence [8] is the displacement of a pathogen strain of low virulence (defined here as the impact of the pathogen on host fitness [9]), by a new, more virulent strain. The mechanisms that facilitate competitive displacement of pathogens are broadly similar to those that act in free-living organisms [7]: (i) exploitation competition, in which the pathogen with the highest rate of transmission pre-empts access to hosts either by killing them [10] or by generating cross-immunity that prevents infection by competitors [11], (ii) direct competition, in which a pathogen suppresses the replication of a co-infecting competitor through mechanisms such as "theft" of proteins by viral genomes [12] or destruction of red blood cells by *Plasmodium *[13], and (iii) apparent competition, in which a pathogen triggers an immune response that is more damaging to co-infecting competitors than to itself [14]. Multiple mechanisms may contribute to displacement concurrently, particularly in vector-borne pathogens where different mechanisms may be enacted in the host and the vector [15].

In the current study we have investigated competitive displacement among strains of mosquito-borne dengue virus (DENV, genus *Flavivirus*, family *Flaviviridae*), the etiological agent of classical dengue fever (DF) and its more severe manifestations, dengue hemorrhagic fever and dengue shock syndrome (DHF/DSS) [16]. DF is an acute febrile illness causing high levels of morbidity but low levels of mortality; DHF/DSS is a capillary leakage syndrome [17,18] with a case fatality rate of up to 14%, although with proper medical care this rate is typically < 1% [19]. DENV is transmitted by mosquitoes in the genus *Aedes*, primarily *Ae. aegypti *and *Ae. albopictus *[18,20]. Mosquito eradication efforts in the mid-1900's reduced the geographic range of DENV to a small number of countries in Southeast Asia, West Africa and the Caribbean. However, subsequent reduction of these efforts, along with changes in global travel patterns and lifestyles, have permitted a resurgence of this virus over the past several decades, and currently 100 million dengue virus infections per year occur in over 100 countries [21-23]. This period has also seen an increase in the severity of dengue disease, and today DENV poses the greatest threat to human health of all arthropod-borne viruses [21-23].

Diversity within DENV lineages falls into three generally-accepted categories [21,24]. At the broadest scale, DENV is comprised of four antigenically-distinct serotypes (DENV1-4). Within the human host, infection with a particular serotype confers lifelong homologous immunity to that serotype and transient heterologous protection against the other three serotypes. However following this period of heterologous protection, sequential infections with different serotypes are associated with enhanced disease [25,26], as documented in Thailand [27,28] and Cuba [29]. The most likely mechanism for this association is antibody-dependent enhancement (ADE), the process by which antibodies against one serotype enhance binding of the other serotypes to FcλR-bearing cells, thereby increasing virus replication and disease severity [25,26]. Within serotypes are embedded genotypes; studies to date indicate that immunity to any genotype within a serotype confers cross-immunity to all other genotypes within that serotype. However genotypes can vary in their tendency to be enhanced by heterologous antibody [30,31] or neutralized by heterologous antibody [26,30]. Additional groupings have been identified within genotypes which have been variously termed subtypes, clades, variants, groups, or strains and which for the sake of clarity are herein termed strains.

Lineage turnover, among serotypes [32-34], genotypes [24,35,36], and strains [37-41] is an increasingly common feature of dengue virus epidemiology. Phylogenetic evidence suggests that some of these turnovers result from evolution of existing lineages [39,42] or from extinction and re-colonization [33,36], while others result from active competitive displacement [24,32,37,38]. Mathematical models of competitive displacement among DENV strains have typically focused on replication in the human host as the driving force for competitive displacement [32,43,44] and have identified the effect of sequential infections by multiple serotypes as a critical determinant of observed patterns of dengue epidemiology. Most have focused on the impact of ADE, reasoning that ADE increases overall virus titer (concentration) in the blood, which in turn increases the likelihood of mosquito infection and subsequent transmission [45], therefore strains with a higher tendency for enhancement are likely to displace those with a lower tendency [44,46,47]. In addition, transient, antibody-mediated cross-immunity between serotypes may also trigger lineage replacement [43].

Mathematical and qualitative models of DENV dynamics that fail to incorporate replication in the mosquito vector will not adequately reflect the complete virus life cycle [15] and may fail to identify critical components of competitive success. In the current study, we have tested the hypothesis that variation in the intrinsic ability of DENV strains to infect their mosquito vector, even when virus titer in the bloodmeal is held constant, may contribute to competitive displacement. We have focused on the spread of a novel DENV3 strain (subtype III, group B) through Sri Lanka in the 1980's and the subsequent displacement of the circulating DENV3 strain (subtype III, group A), a transition that permanently altered the pattern of dengue disease in that country [38,48]. While Sri Lanka had experienced high levels of transmission of all four serotypes of DENV prior to 1989, DHF/DSS was uncommon. This changed dramatically in 1989, when the country experienced a surge in DHF cases that persists to present day [48]. Messer et al. used surveillance data [48] coupled with phylogenetic methods [38] to demonstrate that this emergence of DHF resulted from the displacement of the group A DENV3 strain by the group B DENV3 strain, hereafter termed the native and invasive strains, respectively. Lanciotti et al. [42] first used phylogenetic analysis to investigate the origins of the invasive DENV3 strain in Sri Lanka and concluded that this lineage evolved from the native lineage *in situ *via genetic drift. Messer et al. [38] proposed two alternative hypotheses for the source of the invasive strain, speculating that it may have been introduced from India or East Africa or that it may have been present as a minor population that increased in abundance due to some unidentifed change in the selective environment [38]. Irrespective of the origins of the invasive strain, the rapidity of this lineage turnover during a period of relatively high levels of DENV transmission strongly suggest that the replacement of the native strain resulted from competitive displacement by the invasive strain rather than extinction and re-colonization. Moreover the association of the displacing strain with DHF is consistent with mathematical models that predict that a greater propensity for replication enhancement will confer a competitive advantage. Nonetheless it is important to ask whether the enhanced replication by the invasive strain during DHF is augmented by greater infectivity for mosquitoes, or whether, as has been predicted [49], a trade-off between replication in the primary host and the vector may counteract the advantage of achieving a higher titer in humans.

In this study we measured the ability of the invasive and native DENV3 strains to infect *Ae. aegypti*, the principal mosquito vector of DENV. To be transmitted by a mosquito, DENV must infect and replicated in the midgut disseminate into the hemocoel, infect the salivary glands, and be released into the saliva [50,51]; the first three steps of this process were monitored here. Additionally, to test whether phenotypes in cultured cells might adequately reflect *in vivo *phenotypes, the replication of both the native and invasive DENV3 strains was also tested in several mammalian and mosquito cells in culture. The patterns of infectivity detected *in vivo *give insight into the role of exploitation competition in competitive displacement among DENV strains.

## Results

### Viral fitness in cultured cells

**Figure 1:**
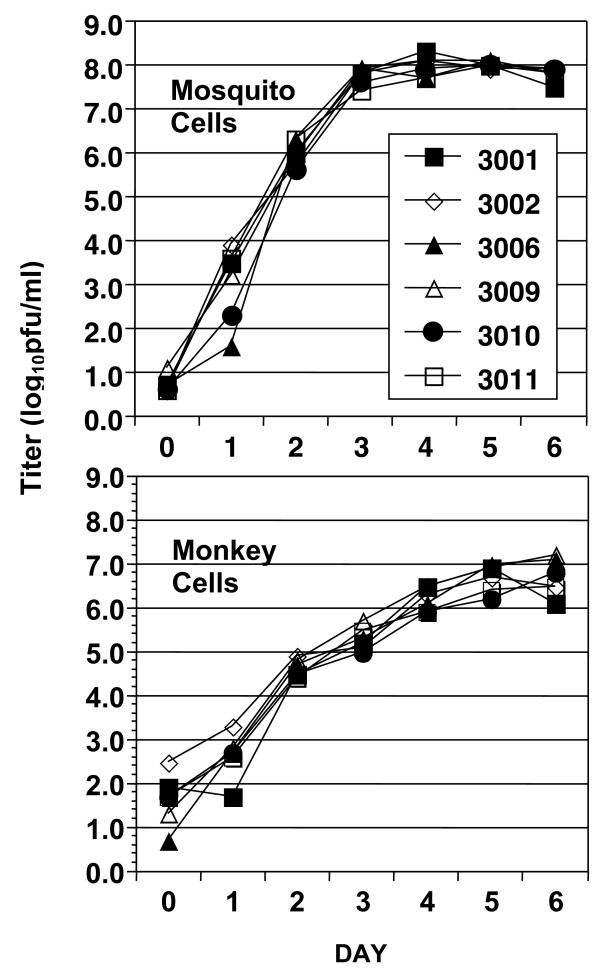
Multicycle replication kinetics of three native (open symbols) and three invasive (filled symbols) DENV3 isolates in mosquito cells (top panel) and African green monkey kidney cells (bottom panel).

The rate of focal spread of a virus through a monolayer of cultured cells, for brevity termed plaque size, can reflect viral fitness [52]. The mean of the 36 plaques measured for each isolate was used as a single value to compare the three isolates of the native strain and three isolates of the invasive strain. Mean plaque size (in mm) of the two strains did not differ in mosquito epithelial (Mean ± 1 se for native = 0.38 ± 0.02, for invasive = 0.39 ± 0.02; student's t-test, df = 4; P > 0.5), human hepatoma (Mean ± 1 se for native = 2.03 ± 0.33, for invasive = 2.61 ± 0.83; student's t-test, df = 4; P > 0.5), or African green monkey kidney cells (Mean ± 1 se for native = 1.36 ± 0.14, for invasive = 0.71 ± 0.36; student's t-test, df = 4; P = 0.07). This analysis was extended to a second measure of viral fitness, multi-cycle replication kinetics, in both mosquito cells and monkey cells. Replication kinetics of the three isolates from each strain were remarkably similar in each cell type (Figure 1). In mosquito cells, neither the mean maximum titer (Mean ± 1 SE: 8.1 ± 0.09 log_10_pfu/ml in the invasive strain, 8.1 ± 0.06 log_10_pfu/ml in the native strain; student's t-test, df = 4, P = 0.77) nor the mean number of days needed to reach that titer (Mean ± 1 SE: 4.5 ± 0.2 days for the invasive strain and 4.8 ± 0.1 days for the native strain; student's t-test, df = 4, P = 0.26) differed between the two strains. Similarly, in monkey cells neither the mean maximum titer (Mean ± 1 SE: 7.0 ± 0.07 log_10_pfu/ml for the invasive strain and 6.8 ± 0.2 for the native strain log_10_pfu/ml; student's t-test, P = 0.57) nor the mean number of days needed to reach that titer (Mean ± 1 SE: 5.2 ± 0.2 days for the invasive strain and 5.5 ± 0.4 days for the native strain; student's t-test, P = 0.64) differed between the two strains. Overall, DENV3 replicated to higher maximum titers at a more rapid rate in mosquito cells than in to monkey cells.

### Virus infectivity for live mosquitoes

**Figure 2:**
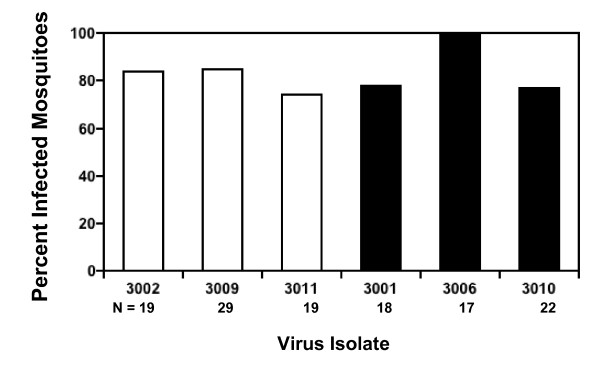
Percent of mosquitoes with detectable virus in the body for each of 3 native (white bars) and 3 invasive (black bars) DENV3 isolates. Sample sizes (N) for each isolate are listed below the isolate number. Native and invasive isolates showed no significant difference in percent of bodies infected.

**Figure 3:**
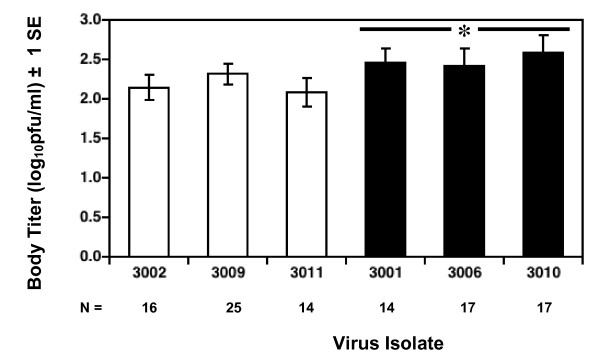
Mean virus titer in the mosquito bodies that had detectable virus in the body for native (white bars) and invasive (black bars) DENV3 isolates. Sample sizes (N) for each isolate are listed below the isolate number. The asterisk above the black bars indicated that invasive isolates produced significantly higher titers on average than native isolates.

**Figure 4:**
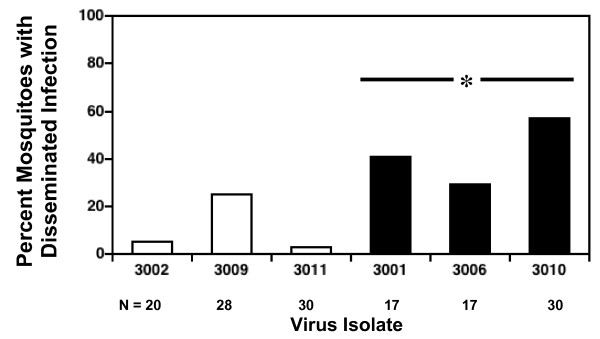
Percent of mosquitoes with virus antigen in the head for each of 3 native (white bars) and 3 invasive (black bars) DENV3 isolates. Sample sizes (N), listed in or above bars, are generally lower for the body than the head because body samples were more often contaminated with fungi. The asterisk above the black bars indicated that invasive isolates infected a significantly higher proportion of heads on average than the native isolate.

**Figure 5:**
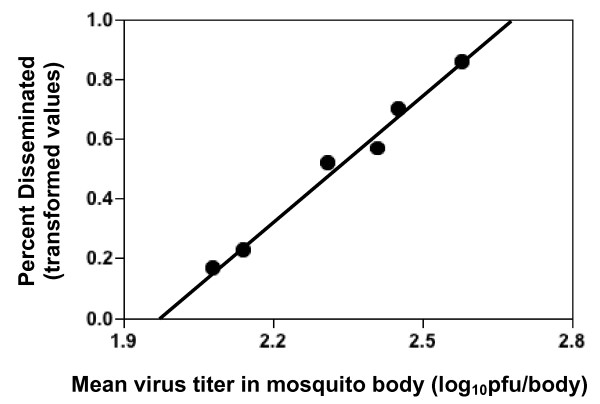
Significant positive regression of percent of heads infected (arcsin-square root transformation of data from Figure 4) on mean virus titer in the body (data from Figure 3) for 6 DENV3 isolates (linear regression, total df = 5, R^2 ^= 0.99, P < 0.001; Y = 1.4X - 2.7). The three lowest points represent the three native DENV isolates.

*Ae. aegypti *were fed on artificial bloodmeals containing comparable, high titers of each of the six DENV3 isolates, and the presence and concentration of virus in the mosquito body were used to measure infection and replication, respectively, while the presence of virus in the head was used to measure dissemination. Native and invasive DENV3 strains did not differ in the percentage of infections generated (Figure 2; median percent infected: native DENV3 = 84, invasive DENV3 = 78; Mann-Whitney U test, N = 6, P = 0.82), however mean virus titer in infected mosquitoes did differ significantly between the two groups (Figure 3, Mean ± 1 se: native DENV3 = 2.2 ± 0.01 log_10_pfu/body, invasive DENV3 = 2.5 ± 0.01 log_10_pfu/body; student's t-test, df = 4, P = 0.02). Moreover, this difference in titer was associated with significant difference in the likelihood of dissemination; invasive DENV3 isolates generated a significantly higher percentage of disseminated infections than native DENV3 isolates (Figure 4: median percent infected: native DENV3 = 5, invasive DENV3 = 41, Mann-Whitney U test, N = 6, P < 0.05). This trend also held true for individual isolates (Figure 5), higher titers in the body were associated with a greater likelihood of virus being detected in the head (linear regression, df = 5, R^2 ^= 0.99, P < 0.001).

To assess the susceptibility of the NIH colony *Ae. aegypti *colony relative to other conspecific populations, *Ae. aegypti *derived from both the NIH and Galveston colonies were fed on artificial bloodmeals containing one of three serotypes of DENV. As shown in Table 1, mosquitoes from the two populations did not differ significantly in their susceptibility to any of these DENV serotypes.

**Table 1:** Susceptibility of *Aedes aegypti *from the NIH and Galveston colony to dengue virus serotypes 1, 3 and 4.

Virus	Colony	No. fed	No. (%) infected^2^	Titer in body of infected mosquitoes [log_10_pfu/body] ± 1 SE^3^	No. (%) disseminated^2^	Titer in head of disseminated infections [log_10_pfu/body] ± 1 SE^3^
rDEN1	NIH	27	4 (15)	3.2 ± 0.5	4 (15)	3.7 ± 0.3
	GAL^1^	30	9 (30)	3.5 ± 0.3	9 (30)	3.4 ± 0.3
rDEN3	NIH	21	10 (48)	3.3 ± 0.2	9 (43)	3.2 ± 0.4
	GAL	11	5 (46)	3.6 ± 0.2	4 (36)	3.1 ± 0.2
rDEN4	NIH	11	10 (90)	4.3 ± 0.2	8 (73)	3.6 ± 0.3
	GAL	12	8 (83)	3.9 ± 0.2	4 (33)	3.8 ± 0.4

1. Galveston colony

2. Pairwise comparisons between the NIH and Galveston colony mosquitoes for each of the three serotypes for both percent infection and percent dissemination detected no significant differences (Fisher's exact test, P > 0.1 for all six comparisons).

3. Pairwise comparisons of the titer of each virus in the body and head of the NIH and Galveston colony mosquitoes also revealed no significant differences between the colonies (Student's t-test, P > 0.08 for all comparisons).

## Discussion

In Sri Lanka in the 1980's, a DENV3 strain (subtype III, group B) that caused a high incidence of severe disease displaced a native DENV3 strain (subtype III, group A) that had been associated with milder disease [38], resulting in an outbreak of severe dengue disease that persists to the present day. While previous studies of the molecular epidemiology of these lineages have documented the pattern of displacement [38,48], they did not investigate the mechanism. The current study tested the hypothesis that exploitation competition, mediated by variation in the ability of the two strains to infect and be transmitted by their mosquito vector, may have contributed to the success of the invasive strain. During exploitation competition, pathogens may monopolize hosts either by killing them or by generating an immune response that prevents infection by competitors. The case fatality rate for dengue disease, even DHF/DSS, is relatively low [19], thus dengue-induced mortality is unlikely to contribute greatly to the dynamics of competition. However, within a serotype, neutralizing antibody generated against one DENV strain will neutralize all others. Thus if two homotypic DENV strains co-circulate in a single host population, each host infected by one of the strains becomes unavailable to the other. Under these conditions the strain with the higher rate of transmission should displace its competitor, in an analogous fashion to the dynamics that result from variation among strains in their tendency to cause ADE [44,46,47].

As a proxy for measuring rate of transmission, we tested the ability of the native and invasive strain to infect, replicate, and disseminate in *Ae. aegypti *mosquitoes, the major vector of epidemic dengue [21]. It has long been recognized that DENV strains may vary in their infectivity for *Aedes *vectors [53,54]. In this study the invasive DENV3 strain infected the same proportion of mosquitoes as the native strain and the difference in replication between the two, although significant, was slight, with the native strain achieving a titer only about twice that of the native strain. Surprisingly, this small difference in replication in the body translated into significantly and substantially greater efficiency of dissemination to the mosquito head than the native DENV3, suggesting that the invasive strain would be transmitted more efficiently than the native strain even if both strains replicated to similar titers in the human host. Studies of two other cases of competitive displacement among flaviviruses have identified variation in vector infectivity as a potential mechanism. First, extensive work by Rico-Hesse and collaborators has shown that the Southeast Asian DENV2, introduced into Cuba in 1981, has subsequently displaced the American DENV2 genotype across most of the Americas. Because the Southeast Asian genotype of DENV2 is associated with DHF/DSS and American DENV2, as a general rule, is not (but note exceptions in Puerto Rico [55] and Niue [56]), this displacement has resulted in large outbreaks of DHF [24]. The SE Asian strain of DENV2 is significantly more infectious for *Ae. aegypti *than the American strain that it has displaced [57-60]. Additionally, the West Nile virus (WNV) 02 strain appears to have displaced the NY99 strain in North America [61]. WNV02 has a shorter incubation period in *Culex *mosquitoes, and consequently more rapid progress to transmission, than WNV NY99 [62,63]. Thus high infectivity for vectors may be a common feature of superior competitors in the flaviviruses and possibly other arboviruses as well.

The differences between the two DENV3 strains in disease association and mosquito infectivity were not reflected by differences in replication in mosquito or mammalian cells in culture, suggesting that phenotypes in culture may not be a reliable indicator of dengue virus phenotypes *in vivo*. Similarly, variation in the ability of WNV NY99 and WNV02 to infect mosquitoes was not evident in the replication of these strains in C6/36 or Vero cells [62].

Three caveats to the findings reported here must be noted. First, populations and species of *Aedes *can vary in their susceptibility to DENV [53,54,64-71], so the susceptibility of *Ae. aegypti *from the NIH colony may differ from that of Sri Lankan populations of this species. Our comparison of the susceptibility of *Ae. aegypti *from the NIH and Galveston colonies for three different DENV serotypes revealed no differences between the two. Nonetheless, it would be worthwhile in the future to test the infectivity of these DENV3 strains in other strains of *Ae. aegypti *and other *Aedes *species. Second, virus may be less infectious when ingested in an artificial rather than a natural bloodmeal [72], though there is no evidence that relative infectivity is affected by the type of bloodmeal used. No tractable animal model that supports high levels of replication of wild type DENV is currently available (although such models are being developed, see [73]), so artificial bloodmeals remain the closest approximation to natural transmission possible for this type of study. Finally, the interpretation that higher levels of infection will result in higher rates of transmission depends upon the assumption that the native and invasive strains have a similar impact on mosquito fitness. Theory suggests that vector-borne pathogens should impose relatively small fitness costs on their vectors, though pathogens of vertebrates may be an exception to this rule [15]. At present, the few studies that have assessed the nature and magnitude of these costs for DENV have all utilized mosquitoes infected via intrathoracic inoculation, a highly efficient but unnatural route of infection. Two of these studies have tested the impact of DENV infection on feeding behavior: Platt et al. [74] reported that DENV infection resulted in a decrease in feeding efficiency, whereas Putnam and Scott [75] reported that it did not. Joshi et al. [76] detected a decrease in survival of *Ae. aegypti *inoculated with DENV relative to controls. In our experience, mosquitoes orally infected with DENV show similar rates of survival compared to mosquitoes fed upon an uninfected bloodmeal (Hanley, unpublished data), but this pattern was not explicitly tested in the current study. Thus, the impact DENV infection on mosquito fitness, and variation among DENV strains in their fitness costs, remain to be investigated.

While the results of this study support the importance of exploitation competition, mediated by variation in vector infectivity, in the displacement of the native DENV3 strain, other mechanisms of competition may also have played a role. For DENV, co-infection of human hosts and mosquito vectors by multiple serotypes and genotypes has been documented [77-80] and in some outbreaks co-infection of mosquitoes is relatively common [81]. Thus direct and apparent competition, both of which require concurrent infection, are possible. Moreover, variation in infection rates and replication within the host remains an important determinant of competitive success. The invasive DENV3 strain, like invasive SE Asian DENV2 [24], was strongly associated with enhanced disease while the native strain was not. At present it is not known whether either strain is associated with severe dengue because it is more prone to enhancement, less prone to cross-neutralization, or intrinsically more virulent than the native strain [30,38,60]. Nevertheless, the idea of DENV strains with a high likelihood of causing severe disease displacing strains that have not caused severe disease in the same setting supports the hypothesis that high levels of replication increase both disease severity and rates of transmission [44,47,82,83]. Elucidating the conditions under which various mechanisms may impact the dynamics of vector-borne pathogens, and determining whether such conditions are met by dengue virus, should provide a fertile area of research. Such studies will be crucial to predicting and controlling the progress of the global dengue pandemic.

## Conclusion

The resurgence of the dengue virus (DENV) pandemic in recent decades has been characterized by increases in both incidence and disease severity. Both may be due in part to the displacement of low virulence DENV strains by higher virulence strains. However the mechanisms that drive strain replacement are not well understood, the impact of intrinsic virulence versus interactions with pre-existing antibody are difficult to disentangle, and the importance of virus-vector interactions has been largely neglected. The current study focuses on the competitive displacement of a native strain of DENV3 in Sri Lanka that was associated with relatively mild disease by a new DENV3 strain associated with severe disease in the 1980's, resulting in an outbreak of dengue hemorrhagic fever that persists to present day. Specifically, we demonstrated that the invasive strain replicates to higher titer and disseminates more efficiently in *Ae. aegypti*, the principal vector of DENV. Thus, in this system, greater replication in the host is coupled to greater replication in the vector, and the synergy of these two phenotypes may explain the competitive success of the invasive strain. These results suggest that the evolution of greater virulence in DENV may not carry the cost of poor infectivity for the vector, and thus the severity of dengue disease may continue to escalate. Since there is currently neither a vaccine nor antiviral therapy available to control the spread of dengue [84], a better understanding of the potential for transmission of highly virulent strains is needed in order to guide surveillance and target control efforts in order to best prevent outbreaks of severe dengue disease.

## Methods

### Viruses and cells

Native DENV3 isolates 3002, 3009 and 3011 correspond to 83SriLan2 [CDC:SK0087], 89SriLan2 [CDC:SK0396] and 85SriLan [CDC:073], respectively in Messer et al. [38]; invasive DENV3 isolates 3001, 3006 and 3010 correspond to 89SriLan1 [CDC:SK0389], 97SriLan1 and 93 SriLan1 [CDC:SK0693], respectively [38]. All six viruses are derived from clinical isolates that were passaged a total of three times in C6/36 cells prior to use in this study. Recombinant viruses rDEN1, rDEN3 and rDEN4 have been utilized as a foundation for dengue virus vaccine development (see Blaney et al. [85] for a review of the origin and passage history of these viruses). Vero cells (African green monkey kidney) [86] were maintained at 35°C in an atmosphere of 5% CO2 in MEM (Invitrogen, Carlsbad, CA) supplemented with 10% fetal bovine serum (FBS), 2 mM Lglutamine (Invitrogen) and 0.05 mg/ml gentamicin (Invitrogen). HuH-7 cells [87] were maintained at 35°C in an atmosphere of 5% CO2 in D-MEM/F-12 (Invitrogen) supplemented with 10% FB, 2 mM L-glutamine, and 0.05 mg/ml gentamicin. C6/36 cells (*Ae. albopictus *epithelial cells) [88] were maintained at 32°C in an atmosphere of 5% CO2 in MEM containing 10% FBS, 2 mM L-glutamine, 2 mM nonessential amino acids (Invitrogen), and 0.05 mg/ml gentamicin.

### Virus phenotypes in cultured cells

Each of the six DENV3 isolates were inoculated at dilutions designed to produce approximately 50 plaques per well onto 80% confluent monolayers of C6/36, Vero, and HuH-7 cells in 6-well plates. Plates were incubated at the appropriate temperature with occasional rocking for 2 hrs and overlaid with 1% methylcellulose supplemented with 2% FBS, 2 mM glutamine and 0.05 mg/ml gentamicin. Plates were incubated for 5 days and plaques were visualized by immunostaining using anti-DENV3 hybridoma cell supernatant as previously described [89]. For each virus-cell type combination, 36 randomly-chosen plaques were measured as previously described [90]. To assess multicycle replication kinetics, each of the six DENV3 isolates were inoculated at a multiplicity of infection of 0.1 onto triplicate confluent monolayers of either Vero or C6/36 cells in 25 mm flasks. Virus was incubated at the appropriate temperature for the cell line for 20 minutes, after which the inoculum was removed and cells washed twice in 3 ml of appropriate media. Each monolayer was then covered in a total volume of 6 ml media. After 5 min, 1 ml of cell supernatant, designated as the Day 0 sample, was removed from each flask, aliquoted into two vials, flash frozen on dry ice and stored at -80°C. Each flask was then replenished with 1 ml appropriate media. Samples were then taken in the same manner at 24-hour intervals for the next six days. Virus titers were determined on monolayers of same cell type as the original substrate for replication by inoculating 24-well plates with serial 10-fold dilutions of cell supernatant. Plates were overlaid with methylcellulose medium, incubated for 5 days, and immunostained as described above.

### Virus infectivity for live mosquitoes

To experimentally measure the infectivity of DENV3 isolates for a natural vector, *Ae. aegypti *mosquitoes from the National Institutes of Health (NIH) colony, see description below, at 5–10 days old were fed on individual artificial bloodmeals containing high titers (7.1–8.1 log_10 _plaque forming units (pfu)/ml) of each of the six DENV3 isolates using previously described methods [90]. There was no significant difference between the mean titer of native (7.7 ± 0.12 log_10_pfu/ml) and invasive (7.6 ± 0.12 log_10_pfu/ml) isolates in the bloodmeals (Student's t-test, df = 4, P = 0.66). Fully engorged females were removed into new containers, fed on cotton pledgets soaked in 10% sucrose and incubated at 27°C, 80% RH for 21 days. Under optimal conditions, dengue virus generally transits from the bloodmeal to the saliva in a period of about 9 days, and recent reports suggest that this time course may be even shorter in certain strains of *Ae. aegypti *[51]. The 21-day incubation period used in this study, which exceeds the median lifespan of *Ae. aegypti *in nature [91] but not in the laboratory [92,93], was chosen to maximize the opportunity for viruses that may replicate relatively slowly to disseminate into the hemocoel, thereby focusing on the ability of each of the six isolates to complete the designated steps in transmission rather than the rate at which they did so. At the end of this period mosquitoes were frozen at -80 C and later dissected. Three samples were taken from each mosquito: (i) To create an archive, legs were removed into a new tube and stored at -80°C, (ii) To assess the efficiency of viral dissemination, the head of each mosquito was removed, squashed on a glass slide and fixed in 100% acetone. Virus antigen was detected in these preparations using an indirect immunofluorescence assay with anti-DENV3 hybridoma cell supernatant as the primary antibody as previously described [45], (iii) To assess overall infection, the remainder of the body was ground using a mortar and pestle in 250 μl Hanks balanced salt solution (Invitrogen) supplemented with 10% FBS, 250 μg/ml amphotericin, 1% ciprofloxacin, and 150 mg/ml clindamycin. Virus titer in each sample was determined by serial titration in C6/36 cell monolayers as described above.

The NIH colony *Ae. aegypti *were derived in 2004 from progeny of the Walter Reed Army Institute of Research (WRAIR) colony. A long period of colony maintenance can affect the susceptibility of mosquitoes to arboviruses [24,94]. To assess the susceptibility of the NIH colony, progeny of another widely used strain of *Ae. aegypti *from the University of Texas, Medical Branch at Galveston were obtained. The Galveston colony was initiated from eggs collected in the Galveston area in 2000–2001 and has been maintained continuously since that time. Groups of mosquitoes derived from each colony were reared under the conditions described above and fed as described above on bloodmeals with titers of approximately 7.0 log_10_pfu/ml of one of three viruses encompassing three different DENV serotypes: rDEN1, rDEN3 or rDEN4. Mosquitoes were dissected as described above, and head and body samples were titered in C6/36 monolayers as described above.

### Statistical analysis

To avoid pseudoreplication, all comparisons of the native and invasive DENV3 used mean plaque size or titer or total numbers infected/uninfected per isolate, e.g. N = 6, for all comparisons. Plaque size, virus titer, maximum titer, and day of maximum titer were compared using a Student's t-test; since each value in the test was a mean of multiple measurements (e.g. 36 plaques/isolates, 3 replicates/growth curve/isolate), parametric statistics were deemed appropriate. All individual titer values for all analyses were log transformed prior to analysis. Percent mosquito bodies and heads infected were compared with a Mann-Whitney U test. To test the effect of mean virus titer in the body on dissemination to the head, values for percent dissemination were first transformed using the arcsine-square root transformation and then a linear regression of these transformed values against the body titer was conducted. Sample sizes were too small to confirm that this transformation rendered the data normal, however a Kendall Rank correlation (not shown) provided qualitatively similar results. In comparisons of the susceptibility of mosquitoes from the NIH and Galveston colonies, for each serotype percent of midguts and heads infected were compared separately using a Fisher's exact test and mean titers were compared using a Student's t-test.

## Authors' contributions

KH conceived of the study, participated in all experiments and statistical analysis, and drafted the manuscript. JN carried out assays of virus titer and infection in all mosquito samples and contributed to the statistical analysis. ES conducted the plaque size and multicycle replication kinetics experiments. SW contributed to experimental design and helped draft the manuscript. CH participated in the design of the study and conducted oral infection of mosquitoes. All authors read and approved the final manuscript.
